# Sleep, anxiety, and depression associated with the quality of life of older adults: a cross-sectional study

**DOI:** 10.15649/cuidarte.5327

**Published:** 2026-05-25

**Authors:** Bianca Pereira de Assis, Laura Cavalaro, Bruno Bomfim Fernandes, Camila Juliana Ferreira Molina, Raiane Caroline Garcia, Aliny de Lima Santos

**Affiliations:** 1 Universidade Cesumar – UNICESUMAR, Maringá, Brazil. E-mail: biiancaassis23@gmail.com Universidade Cesumar Maringá Brazil biiancaassis23@gmail.com; 2 Universidade Cesumar – UNICESUMAR, Maringá, Brazil. E-mail: lauracavalaro9@gmail.com Universidade Cesumar Maringá Brazil lauracavalaro9@gmail.com; 3 Universidade Cesumar – UNICESUMAR, Maringá, Brazil. E-mail: brunobomfimfernandes@gmail.com Universidade Cesumar Maringá Brazil brunobomfimfernandes@gmail.com; 4 Universidade Cesumar – UNICESUMAR, Maringá, Brazil. E-mail: camilamolina.psicologa@gmail.com Universidade Cesumar Maringá Brazil camilamolina.psicologa@gmail.com; 5 Universidade Cesumar – UNICESUMAR, Maringá, Brazil. E-mail: raianercg@gmail.com Universidade Cesumar Maringá Brazil raianercg@gmail.com; 6 Health Sciences Center, Department of Nursing, Universidade Estadual de Maringá (UEM), Maringá, Brazil. E-mail: alsantos@uem.br Universidade Estadual de Maringá Maringá Brazil alsantos@uem.br

**Keywords:** Anxiety, Depression, Sleep, Quality of Life, Aging, Ansiedad, Depresión, Sueño, Calidad de Vida, Envejecimiento, Ansiedade, Depressão, Sono, Qualidade de Vida, Envelhecimento

## Abstract

**Introduction:**

As individuals age, various factors can affect their quality of life, such as changes in sleep patterns and the presence of symptoms of anxiety and depression.

**Objective:**

To analyze the correlation between quality of life and changes in sleep patterns, symptoms of anxiety, and depression in elderly individuals assisted at a Health Unit.

**Materials and Methods:**

This is a quantitative, cross-sectional study conducted with 307 older adults assisted by a Health Unit in southern Brazil. The instruments used were the WHOQOL-BREF and WHOQOL-OLD; the Pittsburgh Sleep Quality Index; and the Hospital Anxiety and Depression Scale. Data collection was conducted at the participants' homes between May and August 2024. Spearman's correlation coefficient was used to analyze the relationships among variables.

**Results:**

A weak correlation was observed between quality of life and sleep quality (r = -0.28 and r = -0.35). In contrast, the correlations with anxiety symptoms (r = -0.47 and r = -0.40) and depression symptoms (r = -0.61 and r = -0.55) were negative and moderate.

**Discussion:**

Domains and facets of quality of life showed negative correlations with the variables analyzed, especially physical and psychological aspects, as well as social participation.

**Conclusion:**

The findings confirm that poor sleep quality and the presence of anxiety and depression symptoms are associated with worse quality of life in older adults, highlighting the importance of continuous monitoring and effective strategies to promote overall well-being.

## Resumen

**Introducción:**

Con el envejecimiento, diversos factores pueden afectar la calidad de vida de las personas, como los cambios en los patrones de sueño y la presencia de síntomas de ansiedad y depresión.

**Objetivo:**

Analizar la correlación entre la calidad de vida y los cambios en los patrones de sueño, así como los síntomas de ansiedad y depresión en personas mayores atendidas en un centro de salud.

**Materiales y métodos:**

Se realizó un estudio cuantitativo transversal con 307 personas mayores atendidas en un centro de salud del sur de Brasil. Se utilizaron los instrumentos WHOQOL-BREF y WHOQOL-OLD, la escala del Índice de Calidad del Sueño de Pittsburgh y la Escala de Ansiedad y Depresión Hospitalaria. La recopilación de datos a domicilio se llevó a cabo entre mayo y agosto de 2024. Se utilizó el coeficiente de correlación de Spearman para analizar la relación entre las variables.

**Resultados:**

Se observó una correlación débil entre la calidad de vida y la calidad del sueño (r = -0,28 y r = -0,35). En contraste, las correlaciones con los síntomas de ansiedad (r = -0,47 y r = -0,40) y depresión (r = -0,61 y r = -0,55) fueron negativas y moderadas.

**Discusión:**

Los dominios y facetas de la calidad de vida mostraron una correlación negativa con las variables analizadas, destacando los aspectos físicos, psicológicos y la participación social.

**Conclusiones:**

Los hallazgos confirman que la mala calidad del sueño, así como la presencia de síntomas de ansiedad y depresión, se asocia con una peor calidad de vida en adultos mayores, resaltando la importancia del monitoreo continuo y las estrategias efectivas que promuevan la mejora del bienestar general.

## Resumo

**Introdução:**

À medida que um indivíduo envelhece, diversos fatores podem afetar a sua qualidade de vida, como alterações no padrão de sono e presença de sintomas de ansiedade e depressão.

**Objetivo:**

Analisar a correlação entre qualidade de vida e alterações no padrão de sono, sintomas de ansiedade e depressão em pessoas idosas assistidas em uma Unidade de Saúde.

**Materiais e métodos:**

Trata-se de um estudo quantitativo, de corte transversal, realizado com 307 pessoas idosas assistidas em uma Unidade de Saúde no Sul do Brasil. Adotaram-se os instrumentos WHOQOL-BREF e WHOQOL-OLD; escala de Índice de Qualidade de Sono de Pittsburgh e a Escala de Ansiedade e Depressão em Hospitais. A coleta domiciliar foi realizada entre maio e agosto de 2024. Utilizou-se o Coeficiente de Correlação de Spearman para analisar a relação entre as variáveis.

**Resultados:**

Observou-se correlação fraca entre qualidade de vida e qualidade do sono (r = -0,28 e r = -0,35). Em contrapartida, as correlações com os sintomas de ansiedade (r = -0,47 e r = -0,40) e depressão (r = -0,61 e r = -0,55) foram negativa e moderada.

**Discussão:**

Domínios e facetas da qualidade de vida apresentaram correlação negativa com as variáveis analisadas, destacando-se os aspectos físicos, psicológicos e a participação social.

**Conclusões:**

Achados confirmam que a má qualidade do sono, assim como a presença de sintomas de ansiedade e depressão, está associada à pior qualidade de vida em pessoas idosas, evidenciando a importância de monitoramento contínuo e estratégias eficazes que promovam a melhoria do bem-estar geral.

## Introduction

The increase in life expectancy has brought with it the challenge of ensuring the maintenance of health and quality of life (QoL) for older adults^1^. The World Health Organization (WHO) defines QoL as the way an individual evaluates their position in life, considering their culture, values, goals, and the environment in which they live^2^. As we age, QoL becomes increasingly determined by the preservation of independence and autonomy, going beyond simply the absence of disease. Thus, an older adult can be considered healthy to the extent that they maintain their functional and cognitive capacity^3^.

Multiple factors can interfere with maintaining the QoL of older adults, such as socioeconomic vulnerabilities like low income and education, as well as low social involvement in collective activities, reinforcing the interference of the emotional dimension^4^,^5^. Thus, isolation, lack of a support network, or changes in daily life habits, whether related to pathological conditions, should be considered when analyzing QoL, since they are also strongly linked to a reduction in this attribute. It can then be inferred that aspects related to QoL have the potential to increase susceptibility to signs and symptoms of depression and anxiety and may also cause changes in sleep patterns^6^-^8^.

Depression is one of the most prevalent mental health conditions among older people^9^-^11^, and there is a recognized inverse relationship between its symptoms and QoL domains, reinforcing the impact of depression symptoms on the well-being of this age group^11^,^12^. Similarly, anxiety has a significant effect on the QoL of older people, compromising their physical, emotional and social health because it is associated with increased isolation, which can lead to the development or worsening of depression^13^.

In turn, sleep plays a fundamental role in human health and, although it is not a natural consequence of healthy aging, it is common for elderly patients to report changes in their sleep patterns^7^. According to Gothe et al.^8^, sleep disorders tend to trigger health problems in older adults, including physical and mental suffering, depressive symptoms and anxiety, and may also be associated with cognitive impairment. Furthermore, studies have confirmed that changes in the quantity and quality of sleep negatively impact on QoL, especially in the physical, psychological, environmental and social relationship domains^5^,^14^.

Although studies link the QoL of this population with signs of depression, anxiety, and changes in sleep patterns, this relationship generally occurs with only some of the elements mentioned, and the number of studies that relate to all these variables in the same population is incipient^5^,^8^,^15^. Given the above, the objective of this study was to analyze the correlation between quality of life and changes in sleep patterns, symptoms of anxiety and depression in elderly people assisted in a Health Unit.

## Materials and Methods

This is a cross-sectional household survey conducted among elderly individuals receiving care at a Primary Health Care Unit in a municipality in the southern region of Brazil. The sample presented is part of a larger, currently ongoing study entitled "Analysis of living conditions and health of elderly individuals assisted in Primary Care in Maringá: a look at the comprehensiveness of care for health promotion".

The primary health care unit (UBS) had three three family health strategies teams that assisted 1660 elderly people. Those registered with the three teams were included in the study, and those who presented cognitive impairments that compromised their understanding of the questions, as tested using the Mini-Mental State Examination^16^; and those who were absent from their homes on at least two attempts, on different days and times, were excluded.

Having obtained lists with the names and addresses of individuals eligible to be included in the study, participants were randomly selected, considering a prevalence of 50%, an estimation error of 5%, and a sample reliability and precision of 95%, plus 5% (16 individuals) for potential losses. The sample consisted of 328 individuals, but after refusals (11) and changes/absence of addresses (10), 307 were effectively interviewed, approached in their homes.

Data collection took place between June and August 2024, during weekdays and Saturdays, in the morning and afternoon. When the individual was not at home, two more visits were made at different times. If it was not possible to conduct the interview, the next person on the list was chosen, with only one substitution allowed.

A structured questionnaire was used, containing four parts: 1. Personal and sociodemographic data (sex, age group, ethnicity, occupation, marital status, and family income) and physical activity; 2. Quality of life; 3. Sleep quality; 4. Symptoms of anxiety and depression. It should be noted that the versions of the scales used were duly adapted and validated for the Brazilian population.

QoL it was verified using the WHOQOL-BREF and WHOQOL-OLD scales. The first test consists of 26 questions to assess a QoL profile in four domains: physical, social, social relationships, and environment. Each item is scored on a scale of 1 to 5, where 1 represents a negative assessment and 5 a positive assessment^17^. The WHOQOL-OLD, on the other hand, is a validated instrument for assessing QoL specifically in older adults, composed of 24 questions divided into six facets: sensory functioning, autonomy, past, present and future activities, social participation, death and dying, and intimacy. Each facet contains four questions, and their answers follow a Likert scale. (1 to 5), with the higher the percentage (closer to 100%) the better the QoL^18^.

The quality of sleep was measured using the Pittsburgh Sleep Quality Index. (IQSP), which consists of 19 questions to assess the participant's sleep quality during the last month. The IQSP was calculated by summing the scores of seven components: subjective perception of sleep quality, sleep latency, sleep duration, sleep efficiency, presence of sleep disorders, use of sleep medications, and daytime sleepiness. Each component received a score between 0 and 3, resulting in a total ranging from 0 to 21 points. It is important to note that the interpretation of the IQSP score is inverse, that is, the higher the total score, the worse the sleep quality. The scores were evaluated as follows: up to 4 points the sleep quality was considered good, between 5 and 10 points poor, and above points indicated the presence of sleep disorders^19^.

Symptoms of anxiety and depression were assessed using the Hospital Anxiety and Depression Scale (HADS), which consists of 14 questions, seven for each subscale: anxiety (HADS-A) and depression (HADS-D). Each question was answered on a scale of 0 to 3, representing the frequency or intensity of symptoms, with the score for each subscale ranging from 0 to 21. The interpretation of the scores for each subscale followed these criteria: 0 to 7 points indicated that the presence of symptoms is unlikely, 8 to 11 points suggested a possible presence of symptoms, and scores between 12 and 21 indicated a high probability of symptoms being present^20^.

The data were analyzed using SPSS version 29.0, employing descriptive statistics – absolute and relative frequency, mean, and standard deviation, and inferential analysis. For numerical variables, data normality was analyzed using the Kolmogorov-Smirnov test and the skewness and kurtosis coefficients. Bootstrapping procedures were also performed (1000 resampling; 95% CI BCa) to obtain greater reliability of the results, to correct possible deviations from normality in the data distribution and differences between group sizes, and to present a 95% confidence interval for the means^21^.

To compare sleep quality, QoL, anxiety, and depression scores as a function of sociodemographic variables, dependent Student 's t-test (two groups) and One-Way ANOVA followed by Tukey 's Post Hoc test (more than two groups) were used. Spearman 's correlation coefficient was used to assess the correlation between QoL domains and facets and sleep quality index, depression scale, and anxiety scores. The magnitude of the correlation was classified as very weak (0.00 to 0.19); weak (0.20 to 0.39); moderate (0.40 to 0.69); strong (0.70 to 0.89); and very strong (0.90 to 1.00). A significant level of p<0.05 and p<0.01 was considered^22^.

In the development of the study, national and international ethical recommendations and standards for research with human beings were followed, and the project was authorized by the Research Ethics Committee of Unicesumar (Opinion: 6.841.861). Participants, after being informed about the objectives and criteria for participation, signed the Informed Consent Form (ICF), in duplicate. The data collected in their entirety are available for free access and consultation on Mendeley Data^23^.

## Results

The study included 307 elderly individuals of both sexes (192 women and 115 men), aged between 60 and 89 years (M=70.16; SD=± 6.66). Table 1 shows the prevalence of elderly individuals aged 60 to 70 years (55.70%), of white ethnicity (63.84%), retired (73.29%), with a partner (61.24%), and with a monthly income of up to 2 minimum wages (69.38%). Regarding lifestyle habits, the majority reported not engaging in physical activity (52.77%).

**Table 1:** Sociodemographic profile and lifestyle habits of elderly participants in the research (n=307). Maringá, Paraná, 2024

Variables	% (n)
Sex	
Female	62.54 (192)
Male	37.46 (115)
Range age	
60-70	55.70 (171)
71-80	35.83 (110)
>80	8.47 (26)
Ethnicity	
Yellow	1.63 (5)
White	63.84 (196)
Indigenous	0.98 (3)
Black	11.07 (34)
Brown	22.48 (69)
Occupation	
Yes	17.59 (54)
No	9.12 (28)
Retiree	73.29 (225)
Marital status	
With partner	61.24 (188)
Without partner	38.76 (119)
Family income	
Up to 1 MW	23.45 (72)
1-2 MW	45.93 (141)
3-4 MW	23.13 (71)
More than 4 MW	6.84 (21)
Practice of physical activity	
Yes	47.23 (145)
No	52.77 (162)

Source: Survey Data, 2024. %: percentile; MW: minimum wage.

**Table 2:** Descriptive analysis of quality of life, sleep quality, anxiety, and depression in elderly users of a Primary Health Care Unit (n=307). Maringá, Paraná, 2024

Variables	Average + DP
QoL Domains	
Physical	3.90 + 0.66
Psychological	3.96 + 0.57
Relations Social	3.83 + 0.58
Environment	3.85 + 0.52
Total score	3.88 + 0.46
QoL Facets	
Functioning of the Senses	4.37 + 0.74
Autonomy	3.82 + 0.62
Activities	3.85 + 0.65
Social Participation	3.84 + 0.65
Death and Dying	4.11 + 0.97
Intimacy	3.98 + 0.83
Total score	4.00 + 0.45
Sleep Quality Domains	
Subjective quality of sleep	0.90 + 0.68
Sleep latency	1.92 + 2.09
Duration in hours	0.56 + 0.79
Typical efficiency	3.00 + 0.00
Sleep disorder	1.17 + 0.55
Use of sleeping pills	0.42 + 0.99
Daytime dysfunction	0.41 + 0.63
Total score ^b^	8.37 + 3.67
Anxiety ^c^	3.78 + 3.94
Depression ^c^	3.66 + 3.34

Source: Research Data, 2024. SD: Standard deviation. a) QoL Domains and Facets – (1 to 2.9 = need for improvement; 3 to 3.9 = fair; 4 to 4.9 = good; 5 = very good); b) Total Sleep Quality Score – (up to 4 points = good; between 5 and 10 points = poor; above 10 points = presence of sleep disorders); c) HADS – 0 to 7 points = unlikely presence of symptoms; 8 to 11 points = possible presence of symptoms; between 12 and 21 = high probability of symptoms).

Table 2 presents a descriptive analysis of QoL, sleep quality, anxiety, and depression in older adults. In the QoL domains, the highest mean score was found in the psychological domain (M=3.96; SD=±0.57), followed by the physical domain (M=3.90; SD=±0.66). Regarding the facets of QoL, the highest mean score was found in the sensory functioning domain (M=4.37; SD=±0.74) and death and dying (M=4.11; SD=±0.97). Regarding sleep quality, a total score of 8.37 (SD=±3.67) was observed, with the lowest score found in the daytime dysfunction domain (M=0.41; SD=±0.63), followed using sleeping medication (M=0.42; SD=±0.99).

Older adults showed an unlikely presence of symptoms of anxiety (M=3.78; SD=±3.94) and depression (M=3.66; SD=±3.34) Table 2.

Significant differences were found in QoL (p=0.036), sleep quality (p=0.010), anxiety (p<0.001), and depression (p=0.017) as a function of sex, showing that men presented better sleep quality and QoL, as well as lower averages of anxiety and depression. It was observed that those aged 80 or older presented lower QoL scores when compared to younger individuals (p=0.019); as did those of mixed race (p=0.013) Table 3.

There was a significant difference in QoL scores obtained using the WHOQOL-BREF (p=0.001) and WHOQOL-OLD (p=0.002), anxiety (p=0.016) and depression (p=0.013) due to the occupational status of older people, indicating that those who were active had a higher QoL score and a lower average of depressive symptoms, while those without active occupations had greater anxiety than retirees Table 3.

Regarding marital status, it was found that older people with a partner have higher QoL scores (WHOQOL-BREF: p=0.001 and WHOQOL-OLD: p=0.016) when compared to those without a partner, and that those without a partner presented a higher depression score (p=0.027). Finally, in the analysis based on income, a lower QoL score (WHOQOL-BREF: p=0.038 and WHOQOL-OLD: p=0.010) was found among those with an income of up to 1 minimum wage as they presented a higher score for depression symptoms (p=0.014) when compared to the others.

**Table 3:** Analysis of the total quality of life score, sleep quality, anxiety, and depression of elderly people using a Basic Health Unit according to their sociodemographic profile (n=307). Maringá, Paraná, 2024

Groups	WHOQOL-BREF	p-value	WHOQOL-OLD	p-value	Sleep quality	p-value	Anxiety	p-value	Depression	p-value
	M + DP		M + DP		M + DP		M + DP		M + DP	
Sex		0.036*		0.070		0.010*		<0.001*		0.017*
Female	3.96 + 0.45		3.85 + 0.47		8.75 + 3.76		4.36 + 4.27		3.95 + 3.52	
Male	4.05 + 0.46		3.93 + 0.45		7.75 + 3.43		2.81 + 3.10		3.16 + 2.96	
Range age		0.019*		0.051		0.287		0.190		0.105
60 to 69 years old	4.04 + 0.42		3.90 + 0.48		8.63 + 3.95		4.16 + 4.28		3.57 + 3.35	
70 to 79 years old	4.00 + 0.46		3.91 + 0.41		7.96 + 3.25		3.52 + 3.72		3.44 + 3.28	
80 or more	3.80 + 0.53^a^		3.70 + 0.54		8.66 + 3.69		3.00 + 2.92		4.77 + 3.38	
Ethnicity		0.051		0.013*		0.260		0.057		0.426
White	4.03 + 0.41		3.93 + 0.44		8.19 + 3.58		3.42 + 3.54		3.42 + 3.15	
Black	4.06 + 0.47		3.98 + 0.46		8.38 + 3.57		3.91 + 4.26		4.06 + 3.47	
Brown	3.86 + 0.54		3.74 + 0.52^b^		9.06 + 4.10		4.87 + 4.74		4.11 + 3.81	
Others	3.93 + 0.33		3.73 + 0.21		7.00 + 2.72		2.75 + 3.24		3.75 + 3.20	
Occupation		0.001*		0.002*		0.824		0.016*		0.013*
Yes	4.19 + 0.32^c^		4.08 + 0.42^c^		8.35 + 3.60		3.81 + 4.37		2.44 + 3.10^e^	
No	3.87 + 0.60		3.79 + 0.58		8.78 + 4.00		5.78 + 4.97^d^		3.85 + 3.76	
Retiree	3.96 + 0.45		3.85 + 0.44		8.33 + 3.65		3.52 + 3.63		3.92 + 3.29	
Marital status		0.001*		0.016*		0.083		0.395		0.027*
With partner	4.06 + 0.45		3.93 + 0.43		8.14 + 3.65		3.83 + 4.20		3.36 + 3.31	
Without partner	3.90 + 0.44		3.81 + 0.46		8.74 + 3.69		3.70 + 3.51		4.12 + 3.36	
Family income		0.038*		0.010*		0.306		0.251		0.014*
Up to 1 MW	3.88 + 0.50^g^		3.73 + 0.44^f^		8.53 + 3.87		4.35 + 4.56		4.71 + 3.50	
1 to 2 MW	4.03 + 0.47		3.90 + 0.48		8.46 + 3.70		3.83 + 4.04		3.28 + 3.24	
3 to 4 MW	4.00 + 0.33		3.96 + 0.39		7.76 + 3.27		3.04 + 3.14		3.20 + 3.19	
More than 4 MW	4.17 + 0.50		4.00 + 0.56		9.33 + 4.04		4.09 + 3.46		3.95 + 3.18	

Source: Survey Data, 2024. M: mean; SD: standard deviation; MW: minimum wage. WHOQOL-BREF: World Health Organization Quality of Life – abbreviated; WHOQOL-OLD: World Health Organization Quality of Life – Quality of life of the elderly. *Significant difference (p<0.05) - Independent Student 's t-test for 2 groups; One-Way ANOVA followed by Tukey 's Post Hoc test for more than two groups among: a) 80 years or older with 60 to 69 years and 70 to 79 years; b) Mixed race with White and Black; c) Yes with No and Retired; No with Retired; e) Yes with Retired; f) Up to 1 minimum wage with 1 to 2 minimum wages, 3 to 4 minimum wages and more than 4 minimum wages; g) Up to 1 minimum wage with more than 4 minimum wages; h) Up to 1 minimum wage with 1 to 2 minimum wages and 3 to 4 minimum wages.

A significant (p<0.05), positive, and moderate correlation was observed between the sleep quality score and the presence of symptoms of anxiety (r = 0.46) and depression (r = 0.41), in addition to a weak negative correlation with the WHOQOL-BREF (r = -0.28) and WHOQOL-OLD (r = -0.35) scores. The WHOQOL-OLD and WHOQOL-BREF showed a significant (p<0.05), negative, and moderate correlation with the presence of symptoms of anxiety (r = -0.47 and r = -0.40, respectively) and depression (r = -0.61 and r = -0.55, respectively). Anxiety showed a significant (p<0.05), positive, and moderate correlation with symptoms of depression (r = 0.56) Table 4.

**Table 4:** Correlation between the total quality of life score, sleep quality, anxiety, and depression of the elderly individuals participating (n=307). Maringá, PR, 2024

Variables	WHOQOL-BREF	WHOQOL-OLD	Sleep quality	Anxiety Symptoms	Depression Symptoms
WHOQOL-BREF	-	0.75**	-0.28**	-0.40**	-0.55**
WHOQOL-OLD		-	-0.35**	-0.47**	-0.61**
Sleep quality			-	0.46**	0.41**
Anxiety Symptoms				-	0.56**
Depression Symptoms					-

Source: Survey Data, 2024. WHOQOL-BREF: World Health Organization Quality of Life – abbreviated; WHOQOL-OLD: World Health Organization Quality of Life – Quality of life of the elderly. ** Significant correlation (p <0.05) – Spearman correlation. **

A weak negative correlation was found between the sleep quality index and the facets of social participation (r= -0.14) and death and dying (r= -0.28), and with the physical (r= -0.36), psychological (r= -0.31), and environmental (r= -0.20) domains. There was a weak negative correlation between the anxiety score and the facets of past, present, and future activity (r= -0.14), social participation (r= -0.25), death and dying (r= -0.25), and the physical (r= -0.38), social relations (r= -0.19), and environmental (r= -0.24) domains, and a moderate negative correlation with the psychological domain (r= -0.41). The strongest correlations were observed in the physical and psychological domains Table 5.

**Table 5:** Correlation between the domains and facets of quality of life and the average obtained in the analysis of the sleep quality index, the depression and anxiety scale, among participating elderly people (n=307). Maringá, PR, 2024

Variables	Sleep quality	Anxiety	Depression
WHOQOL-BREF			
Physical	-0.36**	-0.38**	-0.50**
Psych.	-0.31**	-0.41**	-0.56**
Social Relations	0.02	-0.19**	-0.31**
Env.	-0.20**	-0.24**	-0.38**
WHOQOL - OLD			
Past Activities	-0.10	-0.14*	-0.40**
Soc. Partc.	-0.14*	-0.25**	-0.42**
Death	-0.28**	-0.25**	-0.19**
Intim.	0.03	-0.08	- 0.27* *

Source: Survey Data, 2024. WHOQOL-BREF: World Health Organization Quality of Life – abbreviated; Psych: Psychological; Env: Environment; WHOQOL- OLD: World Health Organization Quality of Life – Quality of life of the elderly. Soc. Partc: Social Participation; Death and Dying; Intim: Intimacy; a. Only facets with a correlation other than zero were retained; *significant correlation (p<0.05);**significant correlation (p<0.01) Spearman 's correlation.

In turn, a moderate negative correlation was observed between the depression symptom score and the facets past, present and future activity (r= -0.40) and social participation (r= -0.42), and with the physical (r= -0.50) and psychological (r= -0.56) domains; as well as a weak negative correlation with the facets death and dying (r= -0.19) and intimacy (r= -0.27), and with the domains of social relations (r= -0.31) and environmental (r= -0.38) Table 5. 

## Discussion

When analyzing the QoL of older adults, it is observed that the highest averages were obtained in the psychological and physical domains, as well as in the facets of sensory functioning, and death and dying. It is understood that, among the aspects that most contributed to a good evaluation of the QoL of the study participants are the ability to maintain independence with the maintenance of senses and physical abilities, since healthy aging is seen as a multidimensional interaction between physical and mental health, independence in daily life, social integration, family support, and economic independence. Similarly, psychological and social integration changes that older individuals undergo can affect their perception of QoL, as confirmed in the present study^24^.

The analysis showed that men presented better QoL and sleep, as well as lower anxiety and depression scores when compared to women. The literature corroborates these findings, indicating that women have a higher prevalence of sleep disorders due to hormonal factors, such as variations associated with the menstrual cycle and menopause, suggesting that sleep quality plays a fundamental role in physical and psychological well-being^25^. Furthermore, psychosocial factors derived from the traditional role of women, such as responsibilities related to family care, also contribute to increased levels of anxiety and depression in elderly women^26^.

It was observed that people over 80 years of age, and those of mixed race, obtained lower QoL scores. With advancing age, the elderly person's body undergoes natural and progressive changes, which increase the predisposition to chronic conditions and loss of functionality, compromising their autonomy and independence, resulting in lower QoL scores^26^. Although aging is an inherent process in human beings, the predominance of low QoL scores among elderly mixed-race people is due to socioeconomic inequalities, more precarious health conditions, and difficulty in accessing and using health services, which leads them to report a worse perception of health compared to white elderly people^27^.

Regarding the economic situation, the present research showed that elderly people with low income (up to 1 minimum wage) obtained lower QoL scores, more symptoms of depression, and worse sleep quality. The relationship between income and health is widely recognized, with recent studies showing that low income can compromise the health of the elderly person, contributing to worse health outcomes and less access to care^4^,^27^. In contrast, those with greater purchasing power have more resources to meet their needs, which provides greater satisfaction in well-being and QoL^28^.

Furthermore, the literature highlights that the presence of a companion is seen as a protective factor, helping to overcome the limitations of aging, reducing social isolation and feelings of loneliness, and contributing to better physical and mental health conditions^13^. This fact is relevant, as active elderly people with companions had higher QoL scores and lower average depressive symptoms. The absence of regular activities can lead to the loss of a structured routine. This scenario results in dissatisfaction with one's own life and emotional impairment, increasing symptoms of anxiety and depression^29^,^30^.

No strong or very strong correlation was found between the variables studied; however, some of them showed moderate strength, which reinforces the interaction between them and the relevance of studying them. The descriptive analysis indicated that only a small percentage of elderly people presented symptoms of anxiety and depression. However, these findings diverge from most research on the subject, which generally reports a high prevalence of these symptoms in the elderly population, influenced by sociodemographic factors and the fact that many participants in these studies reside in long-term care facilities. It is important to highlight that, in the present study, the participants were elderly people residing in the community, which may justify the lower occurrence of these symptoms^11^,^31^.

However, when correlating these variables with QoL, an inverse relationship was observed between the perception of QoL and the presence of symptoms of anxiety and depression among the study participants. National and international studies have obtained similar results^11^,^12^, with particular emphasis on a study carried out in Bahia demonstrating that QoL was negatively and moderately correlated with anxiety and depression, as was the case in the present study^13^.

It is noteworthy that elderly people who are dissatisfied with life and lack the energy to perform daily activities are more likely to present these symptoms. However, anxiety and depression in this population are frequently underdiagnosed and undertreated, as their symptoms may be mistakenly attributed to the natural aging process by the patients themselves and healthcare professionals, resulting in a worsening of the patients' clinical condition^9^.

In turn, sleep quality showed a moderate and positive correlation with symptoms of anxiety and depression, demonstrating that the presence of such alterations can directly interfere with sleep quality among the elderly in this study. The relationship between these variables is considered bidirectional, in which one can be a symptom of the other, and one can potentiate the other. It is common for sleep disorders, such as insomnia or hypersomnia, to be symptoms of anxiety and depression, just as poor sleep quality can contribute to the development of these disorders^32^. Studies indicate that sleep deprivation or a poor subjective perception impairs emotional regulation, leading to increased negative mood, the risk of depressive and anxious symptoms, and reduced motivation and energy for daily activities^11^,^33^.

Regarding sleep quality, it is observed that, even though weak, there was a negative correlation with the domains and facets of QoL, especially those related to physical, psychological, and social aspects. Other studies that analyzed the relationship between these variables obtained the same results, confirming that alterations in the quantity and quality of sleep impact the same domains mentioned^5^,^8^,^14^. With advancing age, it is not uncommon for sleep to become superficial, fragmented, and of shorter duration, leading to insufficient sleep and daytime sleepiness, greater fatigue, less energy for daily activities, and consequently, reducing opportunities for socialization. This creates a cycle of reduced participation in recreational activities and increased social isolation, negatively impacting the QoL of this population^34^.

Furthermore, cognitive difficulties, such as memory lapses and concentration problems, make engaging in conversations and social activities challenging. In addition, mood changes, such as anxiety and depression, associated with poor sleep quality also discourage socialization and engagement in community activities^30^. Therefore, understanding sleep quality is fundamental to improving it and, consequently, promoting a better QoL^14^.

It was found that the presence of anxiety symptoms showed a moderate positive correlation with the presence of depression symptoms, as well as a negative correlation with the psychological domain of QoL. This finding indicates that, although present in a small proportion and without high scores, these symptoms directly impact the QoL and the overall psychological condition of the individuals evaluated. Both disorders show a moderate positive correlation with each other, demonstrating a bidirectional relationship between these variables, that is, both can mutually influence each other^13^. It is important to emphasize that, often, symptoms of depression and anxiety occur together, frequently being aggravated by changes in life circumstances and by the reduction of the social support network. Therefore, it is essential that early interventions aimed at the mental health of the elderly address both depression and anxiety, aiming to offer more comprehensive and effective support^9^.

Furthermore, the presence of depressive symptoms was negatively and moderately correlated with facets and domains of QoL, with emphasis on those of social participation, psychological and physical well-being, reaffirming that isolation, interruption or absence of collective and social activities, as well as the weakening of physical capacity in the elderly, has a strong impact on emotional and cognitive condition. These findings suggest that depression is strongly associated with a worsening of emotional and functional health, significantly influencing the overall perception of QoL. The literature supports that depression in elderly people, often exacerbated by chronic conditions and social isolation, compromises both emotional well-being and physical capacity, generating profound impacts on daily life^31^,^35^.

Older adults with depressive symptoms tend to have lower life satisfaction and a greater propensity for social isolation, factors that can intensify feelings of loneliness and helplessness, worsening the depressive state^13^. Therefore, promoting connection through support networks and community activities becomes crucial to combat the negative effects of isolation, as well as fostering self-esteem, autonomy, social integration, and enabling older adults to take a leading role in their health^24^,^36^.

In general, it is observed that QoL influences and is influenced by sleep quality and the presence of symptoms of anxiety and depression in older adults, especially aspects related to the maintenance of senses and physical capacity, social interactions and participation, and also the psychological condition, so necessary for maintaining autonomy and independence in this population. These findings reinforce the need for public policies that encourage active aging, considering the different needs of this population.

For older people to perceive their QoL in a positive way, it is essential to recognize that active aging is directly linked to well-being and the preservation of physical, mental, and social capacities, allowing their full participation in society. Although aging is a natural process, it should not be synonymous with functional decline but rather seen as a period of adaptation and maintenance of QoL^2^,^24^.

This study presented some limitations, the main one being that it was conducted in only one Primary Health Care Unit in the Southern region of Brazil, which restricts the generalizability of the results. However, the study opens the way for future investigations that explore the relationship between QoL, sleep quality, and symptoms of anxiety and depression. It is recommended that future research be conducted in different Primary Health Care Units, including qualitative approaches, to understand the perceptions and experiences of the elderly population regarding their own QoL and the aspects that influence it.

## Conclusions

The results highlighted the relevance of mental health, with symptoms of anxiety and depression showing a positive correlation with each other and a negative correlation with QoL, especially in the physical, psychological, and social domains. Furthermore, poor sleep quality, considered common among this population, showed weak, but negative, correlations with essential aspects of well-being, such as independence, mood, and socialization. These findings indicate that the tQoL of this population is negatively impacted by poor sleep quality, and that high levels of anxiety and depression symptoms were also associated with a worse QoL.

Given this, it is crucial to reinforce the need for integrated and early interventions that consider the interactions between physical, emotional, and social health in the care of older adults. Primary healthcare professionals, due to their direct contact with this population, must understand that the concept of health in old age goes beyond the absence of pathologies. Therefore, it is essential to raise their awareness of the importance of strategies aimed at preserving independence and autonomy, as well as those that encourage socialization through support networks and community initiatives, promoting overall well-being and a better perception of QoL.
